# Anemia Management in a Jehovah's Witness Patient With Hip Fracture

**DOI:** 10.7759/cureus.106186

**Published:** 2026-03-31

**Authors:** Catarina Morete Cabrita, Andreia Lopes, Francisco Serra Alves, Carla Pereira, Mariana Alves

**Affiliations:** 1 Department of Internal Medicine, Hospital da Horta, Horta, PRT; 2 Department of Clinical Pharmacology and Therapeutics, Santa Maria Local Health Unit, Lisbon, PRT; 3 Department of Orthopedics, Santa Maria Local Health Unit, Lisbon, PRT; 4 Department of Immunohemotherapy, Santa Maria Local Health Unit, Lisbon, PRT; 5 Department of Internal Medicine/Orthogeriatric Unit, Santa Maria Local Health Unit, Lisbon, PRT; 6 Laboratory of Clinical Pharmacology and Therapeutics, Faculdade de Medicina da Universidade de Lisboa, Lisbon, PRT

**Keywords:** anemia/therapy, bloodless medical and surgical procedures, erythropoietin/therapeutic use, jehovah's witnesses, orthogeriatrics

## Abstract

Jehovah's Witnesses are a religious group whose beliefs regarding blood transfusion may pose important clinical and ethical challenges. As such, more patients refuse whole-blood transfusions. Patients with hip fractures often present with anemia, which increases the likelihood of requiring perioperative blood transfusion and increases morbidity and mortality after surgery.

We present here the case report of an 80-year-old female patient, a Jehovah's Witness, with no previous history of thromboembolic events. She was admitted due to a right hip fracture after a fall from a standing height. She revealed pain mobilizing the right lower limb, with external rotation and shortness of the limb. Radiological exams showed a right femoral neck fracture. Lab analysis showed iron deficiency anemia (hemoglobin (Hb) 7.2 g/dL, normocytic and normochromic, serum iron 54.9 µg/dL, ferritin 146 ng/mL, and transferrin saturation (TSAT) 17%; serum folate 5.6 ng/mL and vitamin B12 1229 pg/mL). The patient was treated with intravenous iron supplementation, folic acid, and epoetin beta (30,000 IU twice weekly), to reach a goal of Hb 10 g/dL.

One month after the initial trauma, the patient presented with Hb 11 g/dL and hematocrit 33.2% and underwent hip surgery. On post-surgery day 1, Hb decreased to 8.1 g/dL, hematocrit 24.5%, serum iron 68 µg/dL, ferritin 781 ng/mL, and TSAT 27%, with no blood transfusion needed afterward. One more epoetin beta administration was performed in the immediate postoperative period. The patient experienced a positive clinical and analytical evolution with progressive improvement in physical function and Hb levels above 11 g/dL.

The treatment strategy effectively enabled hip fracture surgery in this patient, improving the patient's outcomes without any postoperative safety issues.

## Introduction

Jehovah's Witnesses number over nine million worldwide, and physicians are increasingly confronted with patients who refuse blood transfusions, posing both clinical and ethical challenges when surgical interventions are required [1].

Hip fractures are frequently associated with anemia, which increases the likelihood of perioperative blood transfusion (approximately 22%) and is associated with increased morbidity and mortality after surgery [2-4].

Perioperative hemoglobin (Hb) refers to Hb levels measured before, during, and after surgery and is a key determinant of oxygen-carrying capacity and surgical risk.

In hip fracture patients, anemia may be exacerbated by acute blood loss, chronic comorbidities, and age-related physiological decline. These factors contribute to poorer surgical outcomes and increased complication rates.

Alternative strategies to optimize perioperative Hb include erythropoiesis-stimulating agents (ESA), iron supplementation, and the correction of modifiable bleeding risks [5,6]. ESA, such as erythropoietin, stimulate erythroid progenitor cells in the bone marrow, thereby increasing red blood cell production and improving Hb levels. Some ESA formulations contain albumin, which may not be acceptable to certain Jehovah's Witness patients.

We report the case of an elderly Jehovah's Witness patient whose surgery was initially postponed due to severe anemia and was successfully performed following preoperative optimization using ESA and iron supplementation.

## Case presentation

We present the case of an 80-year-old Jehovah's Witness patient with metastatic breast cancer (treated in 2018, under palliative care), with hypertension (on chlorthalidone), who was overweight, and with no history of thromboembolism.

The patient was admitted after a fall from a standing height. On admission, she was hemodynamically stable, with no signs of active bleeding. Physical examination revealed pain on mobilization of the right lower limb, external rotation, and limb shortening. Imaging studies confirmed a right subtrochanteric femoral fracture.

Initial laboratory evaluation demonstrated iron deficiency anemia, with Hb 7.2 g/dL, serum iron 54.9 µg/dL, ferritin 146 ng/mL, transferrin saturation (TSAT) 17%, serum folate 5.6 ng/mL, and vitamin B12 1229 pg/mL. Red blood cell indices were normocytic and normochromic. The evolution of the main laboratory parameters from admission to follow-up is summarized in Table 1.

**Table 1:** Serial laboratory parameters from admission to four-week follow-up during perioperative anemia optimization and postoperative recovery. Hb: hemoglobin; TSAT: transferrin saturation

Time point	Hb (g/dL)	Hematocrit (%)	Ferritin (ng/mL)	TSAT (%)	Serum iron (µg/dL)
Admission	7.2	-	146	17	54.9
Preoperative	11	33.2	-	-	-
Postoperative day 1	8.1	24.5	781	27	68
Follow-up (4 weeks)	10	-	1346	12	-

Surgical fixation using a long cephalomedullary nail was indicated; however, the patient declined blood transfusion for religious reasons.

A preoperative optimization strategy was implemented with intravenous ferric carboxymaltose, folic acid supplementation, and epoetin beta (30,000 IU subcutaneously twice weekly for three weeks). Due to the increased thromboembolic risk associated with ESA therapy, epoetin beta was withheld if Hb exceeded 12 g/dL.

**Figure 1:**
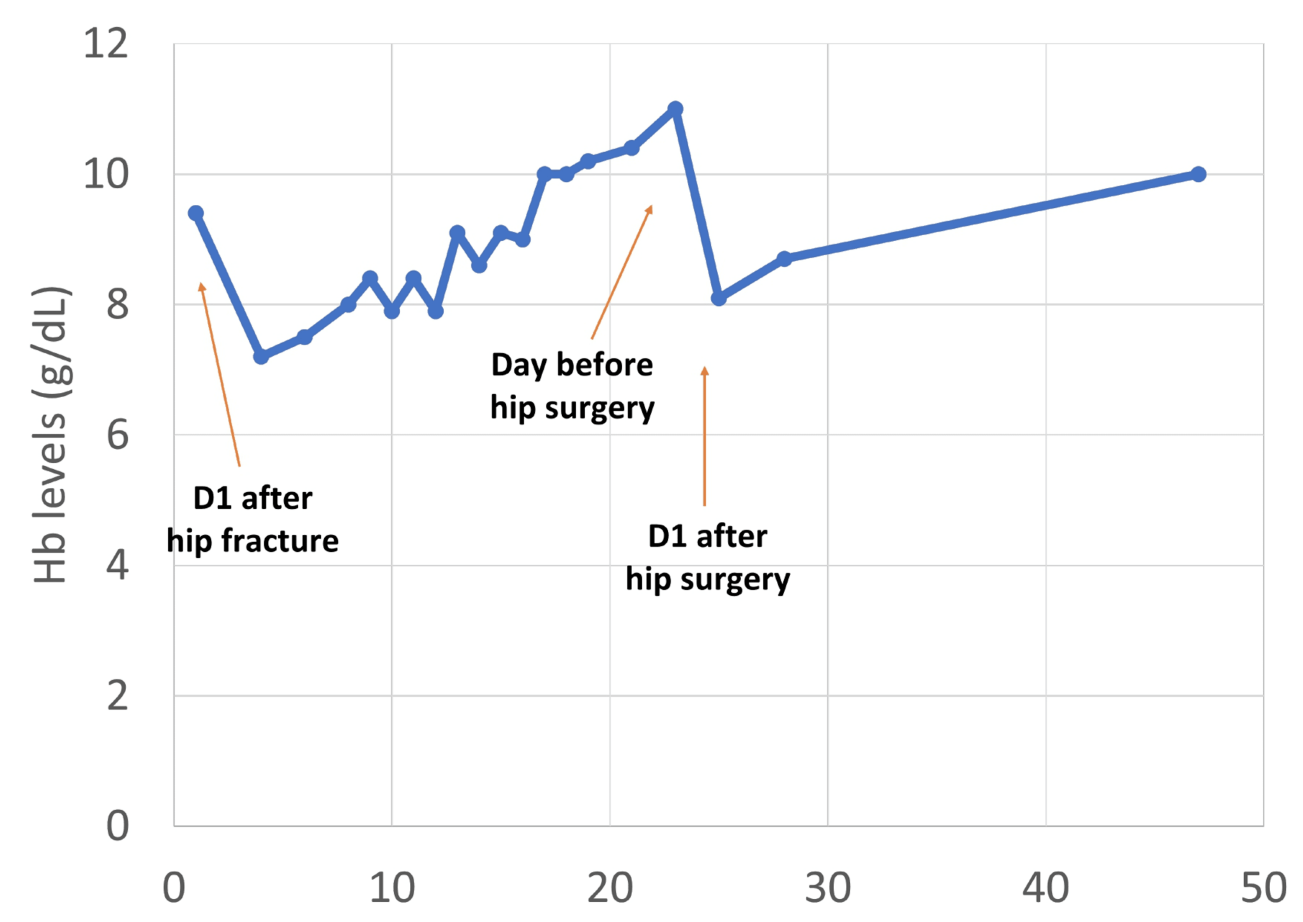
Hb evolution over time during perioperative management. The graph illustrates Hb levels from admission through preoperative optimization, the postoperative period, and follow-up. A marked increase in Hb is observed following treatment with intravenous iron, folic acid, and epoetin beta, enabling surgery without transfusion. A postoperative decrease is observed, followed by gradual recovery. Hb: hemoglobin

After one month, Hb increased to 11 g/dL (Figure 1), representing an approximate 52.8% increase from baseline. This improvement allowed the patient to undergo surgery without a transfusion.

On postoperative day 1, Hb decreased to 8.1 g/dL (hematocrit 24.5%), with serum iron 68 µg/dL, ferritin 781 ng/mL, and TSAT 27%. A final dose of epoetin beta was administered in the immediate postoperative period.

The patient demonstrated progressive clinical and functional improvement with rehabilitation and was discharged with Hb levels above 11 g/dL, without evidence of thromboembolic complications.

At the four-week follow-up, Hb was 10 g/dL, reticulocyte count 3%, ferritin 1346 ng/mL, and TSAT 12%. Given the patient's oncological prognosis, no further treatment or follow-ups were scheduled. She missed her last orthopedics appointment but remained alive.

## Discussion

This case demonstrates that a multimodal approach combining epoetin beta, intravenous iron, and folic acid can effectively optimize Hb levels and enable safe surgical intervention without transfusion in Jehovah's Witness patients.

The increase in Hb observed in this case is in line with the limited literature suggesting that ESA may serve as a useful adjunct in Jehovah's Witness patients undergoing orthopedic and non-orthopedic surgery when transfusion is not an option. However, in some countries, including Portugal, the use of epoetin beta in this setting remains off-label, requiring careful individualized assessment of potential benefits and risks [7,8].

This case also illustrates the potential role of adjuvant therapies in optimizing preoperative anemia and facilitating safe surgical management. Such strategies may be relevant not only for Jehovah's Witness patients but also for individuals who decline transfusion for nonreligious reasons and, more broadly, in settings where minimizing transfusion is desirable given its cost, limited availability, and associated risks, including transfusion reactions and allergic complications [9].

A preoperative Hb target of approximately 10 g/dL appears to provide clinical benefit when ESA therapy is combined with iron supplementation, whereas Hb levels over 11 g/dL have not demonstrated additional benefit and may be associated with increased adverse events [10,11].

ESA options include recombinant erythropoietin and its derivatives (epoetin alfa, epoetin beta, darbepoetin alfa, and methoxy polyethylene glycol-epoetin beta). Epoetin alfa and beta have similar pharmacokinetics, but epoetin beta has a higher molecular weight, different glycosylation, and a longer half-life [12]. In this case, epoetin beta (NeoRecormon©) was used, as it was the only available ESA.

In Portugal, epoetin beta is off-label for patients refusing transfusions due to iron deficiency. The summary of product characteristics (SPC) permits its use in moderate anemia patients participating in autologous donation programs when no preservation processes are available or insufficient [13]. The chosen dosage follows meta-analyses, systematic reviews, and SPC recommendations, without exceeding suggested limits. However, studies on off-label ESA use in Jehovah's Witness report highly variable dosing [14].

Despite its benefits, ESA therapy requires close monitoring, especially off-label. Clinical trials link epoetin alfa and beta to risks of hypertension, myocardial infarction, stroke, venous thromboembolism, vascular access thrombosis, and increased mortality, particularly when Hb levels exceed recommended thresholds (Hb >11 g/dL). Additionally, ESA use in oncologic patients has been associated with shorter survival, increased breast cancer recurrence risk, and higher thromboembolic event rates [15]. Perioperative thromboprophylaxis is advised due to deep vein thrombosis risk [13].

This case highlights the importance of individualized perioperative blood management strategies, which may be applicable not only to Jehovah's Witness patients but also to broader patient populations in whom transfusion carries risk or is undesirable.

## Conclusions

Management of perioperative anemia in patients who refuse blood transfusion remains a significant clinical challenge. This case highlights the importance of individualized perioperative blood management strategies. In selected patients, a multimodal approach combining intravenous iron, folic acid, and epoetin beta may optimize Hb levels and enable hip fracture surgery without transfusion, not only in Jehovah's Witness patients but also in other populations in whom transfusion is undesirable or carries increased risk.

Careful monitoring of Hb levels and thromboembolic risk is essential, particularly when ESA are used outside their primary indications. Although evidence remains limited, this approach may represent a practical option in selected patients. Further studies are needed to establish safe protocols for perioperative anemia management in patients who decline transfusion.
